# Lysine malonylation as a therapeutic target: implications for metabolic, inflammatory, and oncological disorders

**DOI:** 10.1007/s00726-025-03483-0

**Published:** 2025-10-17

**Authors:** Ahsanullah Unar

**Affiliations:** Department of Precision Medicine, University of Campania ‘L. Vanvitelli’, 80138 Naples, Italy

**Keywords:** Lysine malonylation, Malonyl-CoA, Sirtuin 5, Metabolic regulation, Posttranslational modification

## Abstract

Lysine malonylation (Kmal) is an emerging posttranslational modification (PTM) intricately linked to cellular metabolism and disease pathogenesis. This review explores the regulatory mechanisms of Kmal, emphasizing the role of malonyl-CoA as its donor substrate and Sirtuin 5 (SIRT5) as its primary demalonylase. Kmal significantly influences metabolic homeostasis, inflammation, and cancer by modifying key enzymes involved in glycolysis, fatty acid oxidation, and mitochondrial function. In metabolic disorders such as type 2 diabetes and obesity, aberrant malonylation contributes to insulin resistance, lipid accumulation, and oxidative stress. Inflammatory conditions, including sepsis and autoimmune diseases, involve malonylation-driven regulation of immune responses, particularly through GAPDH-mediated cytokine translation. Furthermore, in oncogenesis, malonylation plays a dual role: it suppresses tumor growth by impairing metabolic flux while also being exploited by cancer cells to maintain proliferation. Therapeutic interventions targeting Kmal include SIRT5 modulators, malonyl-CoA metabolism regulators, and small-molecule inhibitors that modulate lysine acylation dynamics. Advances in mass spectrometry and proteomics have expanded our understanding of the biological functions of Kmal; however, its full physiological and pathological significance remains under investigation. Future research should focus on elucidating tissue-specific malonylation patterns and their interactions with other PTMs to refine therapeutic strategies. By integrating metabolic regulation with disease mechanisms, Kmal has emerged as a crucial biochemical modification with broad implications for metabolic, inflammatory, and oncological disorders. Understanding its regulatory network will be pivotal in developing precision medicine approaches aimed at mitigating disease progression and restoring cellular homeostasis.

## Introduction

Lysine malonyl (Kmal) is a recently identified posttranslational modification (PTM) in which a malonyl group (derived from malonyl-CoA) is added to the ε-amino group of a lysine residue (Zou et al. 2023). This modification was first reported in 2011 by Peng et al., who used high-throughput proteomics to identify malonylated proteins in both mammalian and bacterial systems and demonstrated that SIRT5 functions as a lysine demalonylase (Peng et al. 2011). Subsequent systematic mapping further expanded the malonylome across diverse metabolic pathways in mammals (Du et al. 2011). Kmal is evolutionarily conserved, occurring from bacteria to mammals. In *E. coli*, it modifies enzymes involved in energy metabolism and fatty acid biosynthesis (Qian et al. 2016). In cyanobacteria, it regulates photosynthesis and stress adaptation (Ma et al. 2017; Wang et al. 2023). It has also been reported in *Toxoplasma gondii*, where it affects mitochondrial function (Nie et al. 2020). In mammals, malonylation of glycolytic and mitochondrial enzymes underscores its fundamental role in cellular metabolism and homeostasis (Zou et al. 2023). The malonyl moiety carries a negative charge at physiological pH, neutralizing the normally positive charge of lysine (changing its net charge from + 1 to − 1). This dramatic shift in charge can disrupt electrostatic interactions and induce conformational changes in the modified protein. Consequently, malonylation has significant potential to regulate protein function, localization, and interactions, much like phosphorylation or acetylation, but has an even greater impact on protein structure owing to the introduction of an acidic group (Peng et al. 2011; Colak et al. 2015).

The study of Kmal has been empowered by advances in mass spectrometry-based proteomics. Antibody-based enrichment strategies now allow the isolation and identification of malonyl-lysine peptides from complex samples (Tan et al. 2023). With the use of anti-malonyllysine antibodies for immunoprecipitation, thousands of malonylation sites have been mapped in mammalian cells (Du et al. 2011; Colak et al. 2015; Tan et al. 2023). These systematic surveys revealed that more than 20 types of lysine PTMs (including acetylation, succinylation, crotonylation, and malonylation) decorate the proteome, often cooccurring with the same proteins (Zou et al. 2023). The development of sensitive mass spectrometry methods and PTM databases has thus expanded our appreciation for the prevalence of malonylation and its crosstalk with other modifications (Zou et al. 2023). In addition to mass spectrometry, specific anti-Kmal antibodies are also used in Western blots and immunochemistry to detect malonylated proteins in cells and tissues. These technical advances have established Kmal as an important regulator of cellular physiology.

Initial studies revealed malonylation of metabolic enzymes and nuclear proteins, suggesting broad regulatory roles (Zou et al. 2023; Tan et al. 2014). Malonylation of mitochondrial and cytosolic proteins involved in central metabolism, including glycolysis, the tricarboxylic acid cycle, and fatty acid β-oxidation, is especially common (Zou et al. 2023; Du et al. 2011; Tan et al. 2014). By altering enzyme activity or protein–protein interactions, Kmal contributes to tuning metabolic flux and energy homeostasis.

In addition, histones are actively malonylated, and this modification is regulated by SIRT5 (eraser) and the acetyltransferase KAT2A (writer) in a malonyl-CoA–dependent manner; histone malonylation expands the nucleolar area and increases rRNA expression, supporting an epigenetic role for Kmal (Zhang et al. 2023a, b). Notably, global protein and brain histone malonylation increases with age, further linking Kmal to organismal physiology and aging (Zhang et al. 2023a, b).

This review aims to provide a comprehensive overview of lysine malonylation (Kmal) as a critical posttranslational modification, emphasizing its regulatory roles in metabolism, inflammation, and cancer. By examining the biochemical mechanisms underlying Kmal, its impact on metabolic homeostasis, and its involvement in disease pathogenesis, we seek to highlight emerging therapeutic strategies targeting malonylation pathways. Furthermore, we explore the latest advancements in proteomic technologies that facilitate Kmal detection and analysis, underscoring the need for future research to elucidate tissue-specific malonylation patterns and their crosstalk with other posttranslational modifications. Ultimately, this review aims to bridge the gap between fundamental biochemical insights and translational applications, fostering a deeper understanding of Kmal’s potential in disease modulation and precision medicine.

## Lysine malonylation in metabolic disorders

### Malonylation in glycolipid metabolism and type 2 diabetes

Dysregulation of lysine malonylation has been implicated in metabolic syndrome, including glycolipid metabolic disorders (GLMDs) and type 2 diabetes (T2D). T2D is characterized by insulin resistance and hyperglycemia, conditions under which malonyl-CoA levels are often elevated (Zou et al. 2023; Rasmussen et al. 2002; Bandyopadhyay et al. 2006). Malonyl-CoA is the donor substrate for Kmal, so its accumulation provides an increased opportunity for malonylation of proteins. Indeed, a proteomic study by Du et al. revealed 573 malonylation sites on 268 hepatic proteins in diabetic (db/db and ob/ob) mice, significantly more than in normoglycemic controls (Du et al. 2015). These malonylated proteins are enriched in pathways associated with glucose and lipid metabolism. Notably, five key metabolic enzymes that markedly increase malonylation in diabetic mouse livers were validated by immunoblotting: glucose-6-phosphate isomerase (G6PI), 10-formyltetrahydrofolate dehydrogenase (10-FTHFDH), and lactate dehydrogenase A (LDHA) (Du et al. 2015). GSTs were also validated via GSH pull-down, and ALDOB was shown to lose activity upon malonylation in vitro (Zou et al. 2023; An et al. 2004). These enzymes govern critical nodes in glycolysis, gluconeogenesis, and fructose metabolism, and malonylation at specific lysines may modulate their activity. For example, malonylation of LDHA could affect lactate production, whereas malonylation of FBP1 might alter gluconeogenic flux. Through such effects, protein malonylation has been proposed to contribute to hyperglycemia, insulin resistance, and even oxidative stress in diabetic states. Hyperglycemia and excess fatty acids in T2D drive mitochondrial overproduction of reactive oxygen species (ROS); if malonylation impairs the function of antioxidant or metabolic enzymes (as suggested by its widespread increase in diabetic tissues (Zou et al. 2023), this PTM could exacerbate oxidative stress. Conversely, normalizing malonylation levels may improve metabolic resilience. In support of this, in vitro overexpression of malonyl-CoA decarboxylase (MCD, an enzyme that depletes malonyl-CoA) in hepatocytes was shown to reduce malonyl-CoA levels and reverse insulin resistance (Zou et al. 2023; Hu et al. 2005; Sacksteder et al. 1999), suggesting that lowering the malonylation potential can alleviate metabolic defects. Taken together, these data indicate that protein malonylation is elevated under diabetic and dysmetabolic conditions and may causally contribute to metabolic dysfunction.

To further elucidate the mechanistic details and therapeutic implications of lysine malonylation, specifically in type 2 diabetes, the following context and Table 1 summarize key molecular mechanisms, affected proteins, and promising therapeutic interventions.

**Table 1:** Lysine malonylation in type 2 Diabetes, Mechanisms, Targets, and therapeutics

Component	Role in T2D	Effect of malonylation	Therapeutic target/intervention	Key studies
Malonyl-CoA	Substrate for Kmal; accumulates under hyperglycemia, inhibiting CPT1 and FAO	Promotes lipid accumulation, insulin resistance, and mitochondrial dysfunction	MCD activators (reduce malonyl-CoA), ACC inhibitors (ND-630)	(Folmes and Lopaschuk 2007)
G6PI/FBP1	Regulate gluconeogenesis and glycolysis	Disrupts hepatic glucose homeostasis, exacerbating hyperglycemia	SIRT5 agonists (enhance demalonylation)	(Xiong et al. 2003)
LDHA	Catalyzes lactate production in glycolysis	Enhances activity, leading to metabolic acidosis	SIRT5 modulators (restore glycolytic balance)	(Matafome and Monteiro-Alfredo 2024)
SIRT5	Demalonylase; regulates β-cell function and mitochondrial metabolism	Downregulation impairs insulin secretion; overexpression improves β-cell survival	MC3138 (SIRT5 agonist), gene therapy for SIRT5 upregulation	(Wu et al. 2024; Ma and Fei 2018)
CPT2	Facilitates mitochondrial fatty acid oxidation	Malonylation/succinylation reduces activity, promoting cardiac lipotoxicity	SIRT5-mediated desuccinylation (restores FAO)	(Wu et al. 2024)
Pancreatic β-cells	Insulin secretion; regulated by PDX1 transcription	SIRT5 upregulation enhances PDX1 expression via H4K16 deacetylation	SIRT5 activators (improve β-cell function)	(Ma and Fei 2018)

*CPT1*, Carnitine palmitoyltransferase 1; *FAO*, Fatty acid oxidation; *MCD*, Malonyl-CoA decarboxylase; *ACC1*, Acetyl-CoA carboxylase 1; *PDX1*, Pancreatic and duodenal homeobox 1

Key Insights: Malonyl-CoA Accumulation: Drives insulin resistance via lipid deposition and mitochondrial stress. SIRT5 Agonism: Enhances β-cell function and cardiac metabolism, making it a dual therapeutic target. Tissue-Specific Challenges: Systemic SIRT5 modulation risks off-target effects; precision delivery (e.g., β-cell-targeted nanoparticles) is under exploration.

### Malonyl-CoA, fatty acid oxidation, and obesity

Malonyl-CoA is a pivotal metabolite that links glucose and lipid metabolism. It is produced by acetyl-CoA carboxylase (ACC) and plays two major roles: as an essential building block for fatty acid synthesis and as a potent inhibitor of carnitine palmitoyltransferase-1 (CPT1), the gatekeeper enzyme for mitochondrial fatty acid β-oxidation. Through CPT1 inhibition, malonyl-CoA acts as a switch that downregulates fat burning when energy (and acetyl-CoA) is abundant. In obesity and related cardiovascular diseases, chronic overnutrition leads to high ACC activity and malonyl-CoA accumulation, which blunts fatty acid oxidation and can promote ectopic lipid deposition. This mechanism connects malonyl-CoA to obesity-associated insulin resistance and cardiac lipotoxicity (Zou et al. 2023). Kmal provides an additional layer to this regulatory web: elevated malonyl-CoA in obesity/diabetes increases Kmal on enzymes of fatty acid metabolism, potentially reinforcing the blockade of fat oxidation. For example, malonylation of mitochondrial enzymes in β-oxidation could directly diminish their activity, compounding the inhibitory effect of malonyl-CoA on CPT1. Experimental evidence supports the role of malonylation in metabolic control. In hepatocytes and diabetic mouse livers, many enzymes involved in fatty acid oxidation are malonylated (Zou et al. 2023); their malonylation might serve as feedback inhibition when fuel is plentiful. However, in the context of obesity, such inhibition is maladaptive, leading to excess lipid storage and oxidative stress. Conversely, reducing malonyl-CoA levels can be metabolically beneficial: pharmacological inhibitors of ACC (such as ND-630) are known to lower malonyl-CoA and have been shown to increase fat oxidation and improve insulin sensitivity in preclinical models. In cardiomyocytes, lowering malonyl-CoA (by overexpressing MCD or inhibiting ACC) enhances mitochondrial fatty acid uptake and protects against ischemic injury (Table 2). Thus, malonyl-CoA is a double-edged sword necessary for lipid synthesis but is harmful when it is present in excess, and protein malonyl-CoA is emerging as an important mediator of its effects on metabolic tissues.

**Table 2:** Detailed Elucidation of the critical contributions of malonylation to various diseases and the associated mechanisms

Disease or condition	Enzyme or protein involved	Affected cell type	Pathway or mechanism	Biological function and impact	References
Vascular defects	Malonyl-CoA	Endothelial cells	mTOR signaling pathway	Malonyl-CoA influences protein synthesis, cell growth, and proliferation in endothelial cells, which are critical processes in maintaining vascular integrity. Dysregulation contributes to vascular abnormalities	(Zou et al. 2023; Bruning et al. 2018)
Malonic aciduria	Malonyl-CoA Decarboxylase, Malonyl-CoA	Nephrocytes	Fatty acid β-oxidation	Deficiency in Malonyl-CoA decarboxylase leads to excessive accumulation of Malonyl-CoA, impairing fatty acid oxidation and energy production, resulting in metabolic dysfunction	(Zou et al. 2023; Colak et al. 2015; Snanoudj et al. 2021)
Ulcerative Colitis (UC)	Malonyl-CoA	Macrophages	MAPK signaling pathway	Malonyl-CoA-mediated malonylation enhances inflammation by promoting translation of inflammatory cytokines like TNFα, exacerbating tissue inflammation and damage	(Zou et al. 2023; Galván-Peña et al. 2019; Qu et al. 2021)
Diabetes (Type 2 Diabetes)	Malonyl-CoA, Malonyl-CoA Decarboxylase	Liver cells (Hepatocytes)	Glucose and fatty acid metabolism	Elevated Malonyl-CoA levels disrupt normal enzymatic activities involved in glucose and fatty acid metabolism, causing insulin resistance and exacerbating diabetes pathogenesis	(Zou et al. 2023; Du et al. 2015)
Cardiovascular disease	Malonyl-CoA, Malonyl-CoA Decarboxylase	Cardiomyocytes	Fatty acid oxidation and energy metabolism	Regulation of Malonyl-CoA levels is critical for maintaining cardiac metabolic health. Proper control prevents ischemic injury and improves cardiac function by balancing energy metabolism	(Zou et al. 2023; Dyck et al. 2004; Kim 2002)
Neurofibromatosis type 2 (NF2)	Malonyl-CoA, CPT1C	Somatic cells (Somatocytes)	mTOR and lipid metabolism	Malonyl-CoA and CPT1C influence lipid metabolism and cellular growth pathways, significantly affecting tumor cell proliferation and progression in NF2	(Zou et al. 2023; Stepanova et al. 2017)

Adding further evidence to this cardiometabolic link, the authors of that study (Wei et al. 2024) demonstrated that SIRT5-mediated lysine demalonylation of GSTP1 protects cardiomyocytes from pyroptosis in diabetic cardiomyopathy. Hypermethylation of GSTP1 promoted inflammatory cell death and cardiac dysfunction, whereas restoration of SIRT5 activity reduced GSTP1 malonylation and suppressed pyroptotic signaling. These findings highlight that excessive Kmal not only impairs fatty acid oxidation but also directly contributes to maladaptive cardiac remodeling in metabolic disease.

### Impact on glucose metabolism and oxidative stress

In diabetic and high-fat-fed states, malonylation also perturbs glucose metabolism. The aforementioned increase in malonylation of glycolytic and gluconeogenic enzymes (G6PI, ALDOB, LDHA, FBP1, etc.) in diabetes suggests that Kmal may reprogram flux through these pathways (Zou et al. 2023). For example, malonylation of G6PI (which interconverts glucose-6-phosphate and fructose-6-phosphate) could alter glycolytic flow or divert glucose into the pentose phosphate pathway, with consequences for the cellular redox status. Malonylation of FBP1 might suppress gluconeogenesis, contributing to fasting hyperglycemia in individuals with diabetes. Additionally, malonylation of enzymes involved in the TCA cycle and electron transport chain, which has been observed in other studies of Kmal proteomics, can impair mitochondrial respiration, leading to inefficient substrate oxidation and increased leakage of electrons that form ROS. In chondrocytes, high malonylation has been linked to metabolic dysregulation and osteoarthritis in diabetic models, partly via oxidative stress mechanisms (Zou et al. 2023). Moreover (Liu et al. 2025a, b), reported that SIRT5 deficiency in chondrocytes leads to excessive protein malonylation, metabolic reprogramming, and increased oxidative stress, which in turn accelerates osteoarthritis development. Restoration of SIRT5 activity or a reduction in malonyl-CoA levels rescued chondrocyte metabolism and suppressed OA progression. These findings directly implicate Kmal not only in systemic glucose dysregulation but also in cartilage degeneration, providing a mechanistic bridge between metabolic syndrome, redox imbalance, and osteoarticular disease.

**Fig. 1:**
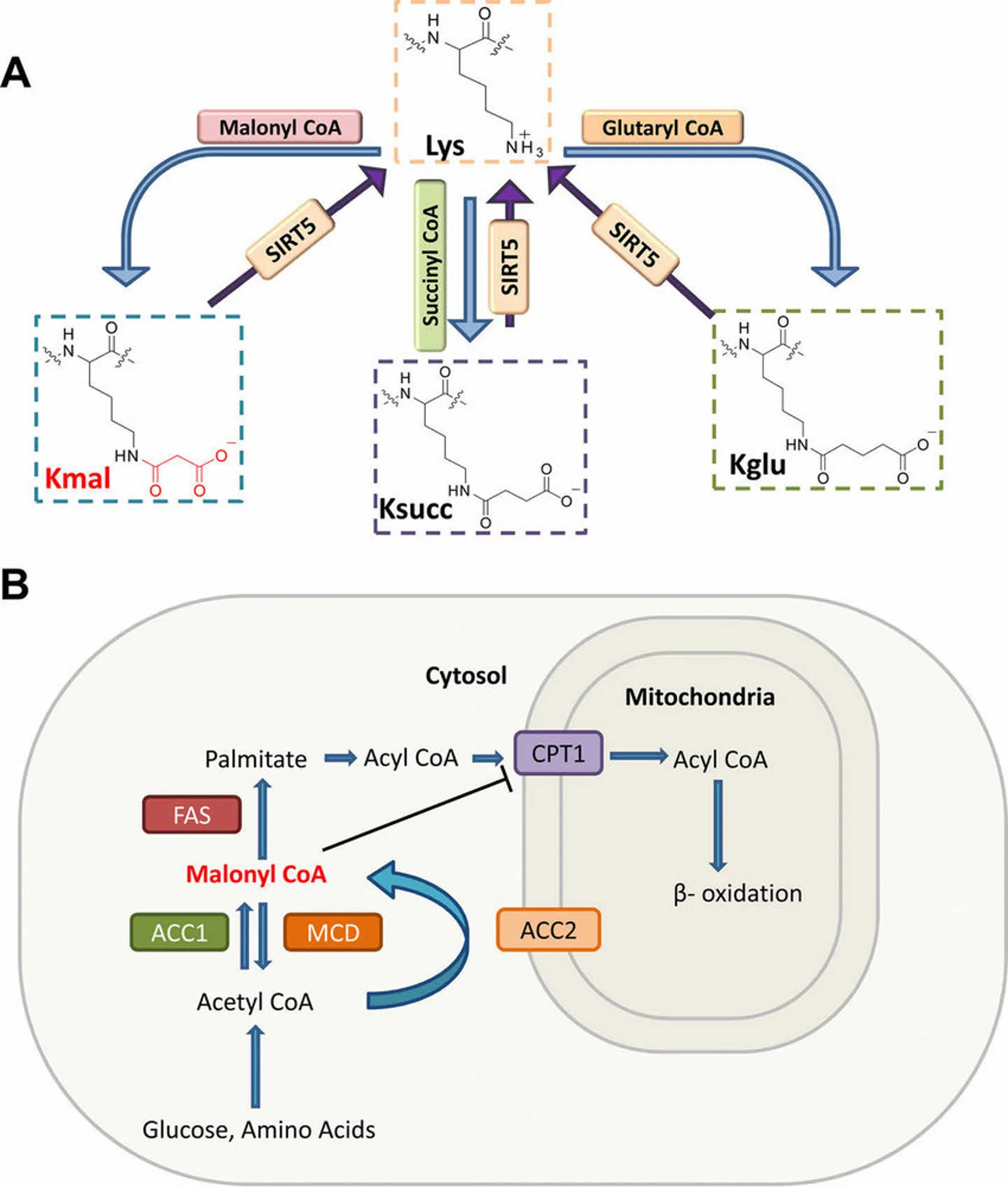
Lysine Malonylation and Malonyl-CoA Biosynthesis. **A** Chemical structures illustrating lysine modifications: malonyllysine (Kmal), succinyllysine (Ksucc), and glutaryllysine (Kglu). Sirtuin 5 (SIRT5) enzymatically regulates these modifications through its demalonylation, desuccinylation, and deglutarylation activities. **B** Schematic representation of malonyl-CoA metabolism. Malonyl-CoA is synthesized from acetyl-CoA primarily by acetyl-CoA carboxylase 1 (ACC1) and is utilized in fatty acid synthesis catalyzed by fatty acid synthase (FAS). Malonyl-CoA levels are reduced by malonyl-CoA decarboxylase (MCD), thus maintaining metabolic balance. Malonyl-CoA also regulates fatty acid oxidation by inhibiting carnitine palmitoyltransferase 1 (CPT1), a mitochondrial enzyme essential for fatty acid transport and subsequent β-oxidation. ACC2, which is localized near mitochondria, specifically influences local malonyl-CoA pools and fatty acid metabolism. (Figure Adopted from (Colak et al. 2015)

There is therefore a plausible chain of events whereby nutrient overload leads to high malonyl-CoA levels, resulting in abnormal protein malonyl-CoA and metabolic enzyme dysfunction, resulting in intermediate accumulation and electron leakage, leading to oxidative stress. By targeting malonylation, disruption of this chain could correct the cellular redox imbalance in metabolic syndrome (Fig. 1).

### Therapeutic potential of targeted malonylation

The reversible nature of lysine malonylation presents opportunities for therapeutic intervention in metabolic disorders. Sirtuin 5 (SIRT5) is an NAD⁺-dependent deacylase that has been identified as the primary *eraser* for malonylation (as well as succinylation and glutarylation) (She et al. 2023). In diabetic models, increasing SIRT5 activity has shown promising results. A recent study reported that hepatic overexpression of SIRT5 in obese diabetic (ob/ob) mice significantly improved glucose tolerance and insulin sensitivity, likely by demalonylating key metabolic proteins and restoring their normal function (Zou et al. 2023). These findings suggest that the hypermalonylation of proteins is deleterious in T2D and that its reversal can ameliorate metabolic abnormalities. Accordingly, SIRT5 and the malonylation status of its substrates are being explored as potential targets for treating type 2 diabetes (Zou et al. 2023). In addition to its role in metabolic regulation, SIRT5 also has therapeutic potential in tissue regeneration (Liu et al. 2025a, b). reported that SIRT5 promotes BMP9-induced osteogenic differentiation by stabilizing HIF-1α protein levels in mouse embryonic fibroblasts. This protective effect against excessive malonylation underscores that enhancing SIRT5 activity not only improves systemic metabolic health but also supports bone formation, linking demalonylation to regenerative medicine and skeletal biology. Small-molecule activators of SIRT5 (to increase malonylation removal) or agents that reduce malonyl-CoA levels in tissues (such as ACC inhibitors or MCD activators) could correct the excessive protein malonylation observed in metabolic syndrome. Conversely, in certain contexts, one might *increase* malonylation to achieve a benefit; for example, transiently increasing malonyl-CoA in the liver might suppress gluconeogenesis via malonylation of gluconeogenic enzymes, helping to lower blood glucose. A nuanced understanding of malonylation dynamics is needed, but these interventions underscore a new therapeutic paradigm. Taken together, these findings position lysine malonylation as a modulatory PTM at the heart of glucose and lipid metabolism, contributing to diseases such as T2D, obesity, and related cardiovascular complications, while offering novel avenues for intervention.

## Inflammation pathways and lysine malonylation

### Macrophage activation and GAPDH malonylation

A striking example of the role of malonylation in inflammation involves immunometabolism. Macrophages activated by bacterial lipopolysaccharide (LPS) rewire their metabolism to support rapid cytokine production. In this state, malonyl-CoA levels rise (partly owing to the shunting of citrate out of mitochondria for fatty acid synthesis), which triggers malonylation of the glycolytic enzyme GAPDH, a moonlighting protein that also binds to mRNAs. The authors discovered that malonylation of GAPDH at lysine 213 is a key step enabling robust tumor necrosis factor-α (TNFα) production in LPS-stimulated macrophages (Zou et al. 2023). In resting macrophages, GAPDH can bind to TNFα mRNA (and other AU-rich element transcripts) and keep it untranslated. Upon LPS stimulation, elevated malonyl-CoA leads to GAPDH K213 malonylation (Zou et al. 2023). This modification causes GAPDH to release TNFα mRNA, freeing the transcript for translation and resulting in a surge of TNFα protein secretion (Zou et al. 2023). In essence, malonylation acts as a switch that converts GAPDH from an “RNA-binding mode” to a “glycolytic mode,” allowing the macrophage to prioritize cytokine production over its normal metabolic restraint. The importance of this mechanism is underscored by the finding that treating mice with recombinant GAPDH protein has an anti-inflammatory effect, reducing TNFα levels in a sepsis model (Zou et al. 2023) (exogenous GAPDH likely sequesters TNFα mRNA without undergoing malonylation). This novel link between metabolism and immunity, termed the “metabolic inflammatory switch,” highlights how a change in a single PTM (Kmal) on a metabolic enzyme can reprogram immune function. This finding provides a molecular explanation for how increased flux through anabolic metabolism (which increases malonyl-CoA) directly feeds into increased inflammatory output. In addition to macrophage-mediated inflammatory control, recent evidence has shown that protein malonylation also regulates adaptive immune responses. The author of that study (Duan et al. 2025) demonstrated that malonate-driven malonylation of STAT6 enhances the generation of memory CD8⁺ T cells, thereby linking metabolic flux through malonyl-CoA to long-term immunological memory. This finding expands the role of Kmal beyond innate immune regulation (e.g., GAPDH in macrophages) to the modulation of adaptive T-cell fate decisions, underscoring its broad impact on host defense mechanisms.

### Sepsis-induced myocardial dysfunction (SIMD) and VDAC2 malonylation

In addition to immune cells, malonylation has been implicated in inflammatory damage to organs. Sepsis, a systemic inflammatory response to infection, often leads to acute myocardial dysfunction characterized by impaired cardiac contractility and bioenergetics. Recent research by She et al. (2023) identified VDAC2, a mitochondrial outer-membrane channel, as a critical malonylation target in sepsis-related heart dysfunction. In a rodent sepsis model, VDAC2 malonylation was significantly elevated in cardiomyocytes, particularly at lysine 46 (She et al. 2023). This modification has pathological consequences: malonylation alters the N-terminal region of the VDAC2 channel, disrupting its conformation and function (She et al. 2023). As a result, mitochondria in septic hearts exhibit a defective membrane potential, excess ROS production, and signs of ferroptosis (an iron-dependent form of cell death) (She et al. 2023). In other words, Kmal of VDAC2 impairs mitochondrial permeability and metabolism, contributing to cardiomyocyte death during sepsis. Crucially, the study demonstrated that these effects are driven by malonyl-CoA accumulation and are reversible. Malonyl-CoA was identified as the primary inducer of VDAC2 malonylation in sepsis, and treatments that lowered malonyl-CoA or blocked the malonylation site on VDAC2 were shown to be protective (She et al. 2023). Specifically, inhibiting ACC2 (the cardiac isoform of acetyl-CoA carboxylase) with the drug ND-630 reduced malonyl-CoA levels, which in turn diminished VDAC2 malonylation, restored mitochondrial function, and alleviated ferroptotic cell death in septic hearts (She et al. 2023). Additionally, a mutant VDAC2 that could not be malonylated (K46Q mimicking a permanently unmodified state) was resistant to sepsis-induced dysfunction (She et al. 2023). These interventions resulted in improved cardiac contractility and reduced markers of myocardial injury in septic animals. Together, these findings position Kmal as a mediator of sepsis pathology: it links the inflammatory surge (which alters metabolism) to organ damage via mitochondrial dysregulation. Targeting this pathway, for example, by enhancing SIRT5 activity to eliminate malonyl-CoA decarboxylase or by supplying malonyl-CoA decarboxylase to consume malonyl-CoA, could be a therapeutic strategy to protect the heart (and possibly other organs) in sepsis. Indeed, overexpressing SIRT5 in cardiomyocytes has been shown to reverse VDAC2 hypermalonylation and reduce sepsis-induced ferroptosis (She et al. 2023), further validating this approach.

Importantly, similar protective mechanisms involving SIRT5 have been observed in chronic cardiac disease (Wei et al. 2024). demonstrated that SIRT5-dependent demalonylation of GSTP1 suppresses cardiomyocyte pyroptosis in diabetic cardiomyopathy. By preventing excessive malonylation of GSTP1, SIRT5 preserves redox balance and reduces inflammatory cell death in diabetic hearts. These findings extend the role of malonylation beyond acute sepsis-induced dysfunction, showing that aberrant Kmal contributes to both acute and chronic cardiac injury and positioning SIRT5 as a therapeutic safeguard across diverse cardiac pathologies.

### Malonylation and inflammation resolution therapies (SGLT2 inhibitors)

The connection between metabolism and inflammation via malonylation suggests that metabolic drugs might have unappreciated anti-inflammatory effects by modulating Kmal. A prominent example is the class of sodium‒glucose cotransporter 2 (SGLT2) inhibitors (e.g., empagliflozin and dapagliflozin), which are used for T2D and reduce inflammation and improve cardiovascular outcomes in patients. Recent studies have indicated that SGLT2 inhibitors alleviate inflammation in part by reducing protein malonylation in immune cells. In a rat model of cardiac inflammation (induced by angiotensin II), SGLT2 inhibition led to increased ketone body levels (a known effect of this drug class) and a concomitant decrease in ACC1 expression in epicardial adipose tissue (She et al. 2023; Li et al. 2023). A lower ACC1 activity means that less acetyl-CoA is converted to malonyl-CoA; accordingly, malonyl-CoA concentrations decrease in treated animals. This resulted in a measurable reduction in GAPDH malonylation in inflammatory cells from epicardial fat (Li et al. 2023). Moreover, the levels of proinflammatory cytokines (such as TNFα and IL-6) in the tissue were significantly reduced. Mechanistic in vitro experiments in macrophages confirmed this pathway: adding the ketone body β-hydroxybutyrate (to mimic SGLT2 inhibitor metabolism) downregulated ACC1, lowered malonyl-CoA and GAPDH-Kmal, and blunted LPS-induced TNFα and IL-1β production (Li et al. 2023). When ACC1 was experimentally knocked down, similar anti-inflammatory effects were observed, and importantly, these effects were reversed by malonyl-CoA supplementation (Li et al. 2023). These findings establish that the ACC1–malonyl-CoA–GAPDH malonylation axis is a key mediator of inflammation. The in vivo outcome was that SGLT2 inhibitor–treated rats had significantly less inflammatory cell infiltration and fibrosis in the heart, which was correlated with reduced GAPDH malonylation (Li et al. 2023). In summary, SGLT2 inhibitors exemplify how the modulation of cellular metabolism can intersect with PTMs to influence immune responses. By downregulating malonyl-CoA production, these drugs inadvertently reduce pathological malonylations of proteins such as GAPDH, thereby dampening excessive inflammation. This mechanistic insight not only explains some of the cardioprotective and anti-inflammatory benefits of SGLT2 inhibitors observed clinically but also suggests that other metabolic interventions (e.g., ketogenic diets or ACC inhibitors) might be repurposed to control inflammation through the targeting of malonylation. These findings highlight lysine malonylation as a novel pharmacological node for anti-inflammatory therapy.

## Lysine malonylation in male fertility and reproductive health

Emerging research on reproductive biology indicates that lysine malonylation plays a role in sperm function and male fertility. Spermatozoa are highly specialized cells with a unique metabolism geared toward motility and fertilization. They rely on both glycolysis (particularly in the flagellum) and oxidative phosphorylation (in the midpiece mitochondria) to generate ATP for movement. Recent high-resolution proteomic analyses of human sperm have revealed that lysine malonylation, along with other acyl-lysine modifications, is widespread on sperm proteins and may modulate these crucial energy-producing pathways (Tian et al. 2024). In a comprehensive study (Tian et al., 2024), researchers studied four lysine acylation pathways in the sperm of fertile and nonfertile men: classical acetylation, succinylation, and crotonylation (Tian et al. 2024). They identified hundreds of sites of malonylation on sperm proteins, many of which are enzymes involved in glycolysis (e.g., glyceraldehyde-3-phosphate dehydrogenase, enolase) and oxidative phosphorylation (electron transport chain components), as well as structural proteins important for sperm motility (such as the dynein arms of the flagellar axoneme). This enrichment of metabolic enzymes by Kmal suggests a regulatory role: by potentially toggling the activity of these enzymes, malonylation could influence how efficiently a sperm produces ATP and thus its swimming capability. For example, malonylation of GAPDH-S (the sperm-specific isoform of GAPDH tethered to the fibrous sheath of the flagellum) might inhibit its glycolytic activity, leading to lower ATP generation in the flagellum and reduced motility. Similarly, malonylation of mitochondrial proteins can affect sperm respiratory efficiency. These findings are supported by observations in infertile populations; specifically, men with asthenozoospermia (a condition of reduced sperm motility) exhibit altered patterns of lysine acylation in their sperm proteins compared with normozoospermic (fertile) men (Tian et al. 2024). Proteomic comparisons revealed that asthenozoospermic sperm presented differential levels of malonylation (and succinylation) of key enzymes involved in energy metabolism, suggesting that hyper or hypomethylation at certain sites might underlie motility defects. Although the exact functional consequences of each modification are still being elucidated, aberrant malonylation clearly correlates with poor sperm performance.

In addition to motility, malonylation may influence sperm viability and the spermatogenic process. Lysine malonylation has been detected in proteins involved in spermatogenesis in the testes, such as histones and chromatin-associated proteins that undergo major rearrangements during sperm development. Improper PTMs of these proteins can result in aberrant chromatin packaging or DNA damage in sperm, potentially leading to male infertility. There is evidence that disruptions in sirtuin enzymes, which regulate many types of lysine acylation, are linked to reproductive issues. In particular, SIRT5, the NAD⁺-dependent enzyme that removes malonyl, succinyl, and glutaryl groups from lysines, is expressed in male germ cells and in mature sperm. Studies have shown that downregulation of SIRT5 is associated with impaired spermatogenesis and decreased sperm quality (Bello et al. 2022). In mouse models, the loss of SIRT5 can lead to the accumulation of malonylated mitochondrial enzymes, which might disrupt the energy supply during spermatid maturation and sperm capacitation. The authors proposed that the dysregulation of mitochondrial sirtuins (SIRT4 and SIRT5) in the testes of obese or diabetic animals contributes to infertility by altering the acylation patterns of proteins critical for germ cell development (Bello et al. 2022). Consistent with this, obese men often have lower SIRT5 levels in the testes and suffer from lower sperm counts and motility, possibly due to excessive malonylation/succinylation of metabolic enzymes in sperm. Recent studies have further highlighted how systemic metabolic hormones influence reproductive health via malonylation (Ke et al. 2024). reported that leptin, which is elevated in obesity, downregulates SIRT5 and increases β-catenin malonylation, thereby impairing BMP9-induced osteogenesis in mesenchymal stem cells. Although this work focused on bone biology, it underscores a broader principle: metabolic and hormonal cues can repress SIRT5 activity, drive pathological malonylation of key regulatory proteins, and impair tissue function. These findings suggest that obesity-linked hyperleptinemia may also affect reproductive tissues through similar SIRT5–malonylation pathways, providing a mechanistic link between metabolic syndrome, infertility, and abnormal gamete quality (Ke et al. 2024). Conversely, high SIRT5 activity (or low malonylation) seems to be favorable for sperm function, ensuring that enzymes such as GAPDH-S and pyruvate kinase M2 (another flagellar glycolytic enzyme) remain active to produce ATP. This balance suggests potential diagnostic and therapeutic angles: measuring the malonylation status of specific sperm proteins could serve as a diagnostic biomarker for certain male infertility conditions (e.g., a high level of malonylation of sperm glycolytic enzymes might indicate asthenozoospermia). Therapeutically, one could envision interventions to adjust malonylation levels in the male reproductive tract. For example, antioxidant or metabolic therapies that alter malonyl-CoA levels (such as L-carnitine or omega-3 supplements, which impact fatty acid oxidation) might indirectly reduce pathological malonyl-CoA levels and improve sperm motility. Additionally, if specific malonylation sites critical for sperm function are identified, targeted drugs or even dietary interventions could be developed to modulate those PTMs. While such treatments are still speculative, the clear linkage of Kmal with sperm metabolism and fertility is a promising avenue for further research.

In summary, lysine malonylation has emerged as an important PTM in the male reproductive system that impacts sperm energy metabolism and quality. Recent leptin–SIRT5–malonylation findings emphasize that reproductive health is tightly coupled with systemic metabolic status, reinforcing malonylation as a nexus between metabolism, fertility, and endocrine regulation (Ke et al. 2024). This study not only advances our understanding of male fertility at the molecular level but also opens new possibilities for diagnosing sperm defects and enhancing male reproductive health through PTM-targeted strategies.

## Renal disease and lysine malonylation

Chronic kidney disease (CKD) and end-stage renal disease (ESRD) are accompanied by systemic metabolic and inflammatory disturbances, and recent evidence implicates aberrant lysine malonylation in their pathogenesis. In CKD, patients often exhibit chronic inflammation, endothelial dysfunction, and coagulation anomalies (particularly platelet dysfunction) (Tan et al. 2023). The authors undertook an integrated proteome and malonylome analysis of peripheral blood mononuclear cells (PBMCs) from ESRD patients, providing the first global look at protein malonylation in kidney disease (Tan et al. 2023). The study identified 793 differentially expressed proteins (DEPs) and 12 differentially malonylated proteins (DMPs) (encompassing 16 specific lysine-malonyl sites) when comparing ESRD patients to healthy controls (Tan et al. 2023). Bioinformatic pathway enrichment revealed that the Rap1 signaling pathway and the platelet activation pathway were significantly impacted in ESRD, with several components of these pathways showing altered abundance or malonylation (Tan et al. 2023). Rap1 is a small GTPase that, in platelets and leukocytes, mediates inside-out signaling to integrins (crucial for cell adhesion and aggregation). Notably, two proteins have drawn particular attention: talin-1 (TLN1) and β-actin (ACTB). Talin-1 and actin are key players in integrin activation, and the cytoskeleton changes during platelet aggregation and leukocyte adhesion. In ESRD PBMCs, *talin-1 expression is downregulated at the protein level*,* and talin-1 is malonylated at lysine 2024* (Tan et al. 2023). This site (K2024) lies in the talin C-terminal rod domain, which is important for its interaction with Rap1 effectors and integrin β tails. Malonylation at talin-1 K2024 is predicted to alter talin’s conformation or binding affinity, thereby impeding the normal Rap1–RIAM–talin signaling axis required for integrin activation (Tan et al. 2023). In functional terms, such a modification could reduce the ability of Rap1 to induce platelet αIIbβ3 integrin activation (necessary for platelet clumping) or diminish the leukocyte integrin (β2) activation needed for immune cell extravasation (Tan et al. 2023). This finding is consistent with clinical observations: ESRD patients often have a paradoxical combination of bleeding tendency (from platelet dysfunction) and an elevated risk of thrombosis/inflammation, partly due to uremic toxin effects on these cells. The malonylation of talin-1 provides a novel molecular mechanism that could contribute to this phenomenon. Additionally, β-actin (ACTB), a fundamental component of the cytoskeleton, was found to be differentially malonylated in ESRD (Tan et al. 2023). Actin dynamics are essential for both platelet shape changes during activation and for immune cell motility; malonylation of actin might affect filament formation or interaction with actin-binding proteins, further contributing to cellular dysfunction in ESRD. Importantly, the affected pathways (Rap1 signaling, platelet activation, and regulation of the actin cytoskeleton) are interrelated and central to vascular homeostasis (Tan et al. 2023). The enrichment of malonyl groups in these pathways suggests that Kmal could be an upstream regulator of endothelial and immune abnormalities in kidney disease.

From a biomarker perspective, the malonylation profile of ESRD patients could have diagnostic or prognostic value. Tan et al. defined a set of malonylated peptides unique to ESRD patients’ PBMCs (Tan et al. 2023). As a proof of concept, one could imagine a ‘malonylation signature’ (for example, high Kmal at talin-1 K2024 and actin, plus others) that correlates with CKD progression or complications. This might be assessed via mass spectrometric analysis of blood cell proteins or perhaps by more accessible means if antibodies to specific malonyl epitopes are developed. Because CKD is often a progressive disease, tracking changes in such a malonylation signature could indicate worsening inflammation or the imminence of complications such as cardiovascular events. In terms of therapy, targeting malonylation in CKD is an intriguing new idea. If malonylation of talin-1 contributes to platelet dysfunction, could we reduce that modification to improve hemostasis? One approach might be to increase the activity of SIRT5 in blood cells, thereby eliminating malonyl groups from talin and actin. Recent work by Baek et al. (2023) provides strong evidence for this strategy: they demonstrated that SIRT5 protects against diabetic kidney disease by suppressing non-mitochondrial protein malonylation in renal cells. In SIRT5-deficient models, hypermalonylation accumulated in the cytoplasm and nucleus, driving metabolic dysfunction and inflammation, whereas SIRT5 overexpression restored proteostasis and improved renal outcomes. This highlights SIRT5 as a therapeutic safeguard that restrains malonylation beyond mitochondria, directly linking Kmal dysregulation to kidney pathology.

Another approach is indirect: since malonyl-CoA is the source of Kmal, strategies that normalize cellular metabolism in uremia (dietary interventions, vitamin B7/biotin supplementation to modulate carboxylases, etc.) might lower malonyl-CoA accumulation and thus protein malonylation. Notably, uremic patients often have altered lipid and glucose metabolism, which could affect malonyl-CoA production. Additionally, Rap1 signaling itself could be targeted, for example, by stabilizing talin-1 or enhancing downstream signaling to compensate for any inhibitory effects of malonylation. While these ideas are speculative, the identification of malonylation in CKD has opened a fresh line of inquiry. In summary, malonylation appears to be involved in the pathophysiology of renal disease by modifying proteins in critical cell signaling pathways (rap1/integrin in platelets and immune cells). The integration of patient malonylome data with mechanistic studies in DKD underscores that both systemic and cell-intrinsic malonylation events shape kidney dysfunction. Increasing SIRT5 activity or modulating malonyl-CoA metabolism represents a promising avenue to correct these imbalances. Its status in patients correlates with disease-related dysfunctions, highlighting its potential as both a biomarker of CKD/ESRD progression and a therapeutic target to correct metabolic and immune imbalances in these conditions (Tan et al. 2023).

## Cancer and lysine malonylation

Lysine malonylation has a complex, context-dependent influence on cancer biology and plays dual roles in tumor promotion and suppression. Cancer cells extensively reprogram their metabolism, a phenomenon often referred to as the Warburg effect (aerobic glycolysis), along with increased glutamine utilization and fatty acid synthesis. Because malonyl-CoA is a central metabolite for lipid biosynthesis and a regulator of mitochondrial fuel use, it is not surprising that malonyl-CoA is emerging as an important modulator of tumor metabolism. One facet of malonylation in cancer is its tumor-suppressive potential via metabolic inhibition. As discussed earlier, malonylation can inhibit key metabolic enzymes; for example, malonylation of GAPDH diminishes its glycolytic activity (Zou et al. 2023; Tan et al. 2023), and malonylation of enzymes in the TCA cycle or β-oxidation can slow those pathways. Tumor cells often rely on the maximal activity of these pathways to generate biomass and energy; thus, excessive malonylation can be detrimental to their growth. This principle mirrors recent findings in noncancer diseases such as osteoarthritis (Liu et al. 2025a, b), diabetic cardiomyopathy (Wei et al. 2024), and kidney disease (Baek et al. 2023), where hypermalonylation suppresses energy metabolism and drives tissue dysfunction. The consistency of this metabolic brake across both tumor and nontumor settings underscores why cancer cells upregulate SIRT5 to erase malonylation and maintain metabolic flexibility. Evidence for this phenomenon comes from studies of SIRT5, an NAD⁺-dependent demalonylase. SIRT5 is frequently overexpressed in cancers, presumably to eliminate growth-inhibitory acylations (such as Kmal and Ksucc) and keep metabolism hot. In cell culture and animal models, the loss of SIRT5 has been shown to impair cancer cell proliferation. For example, SIRT5 knockout (or knockdown) in lung cancer cells led to increased malonylation of glycolytic enzymes and significantly reduced tumor growth both in vitro and in vivo. This aligns with the idea that malonylation accumulation (due to a lack of SIRT5 “eraser” activity) pushes metabolic enzymes into a less active state, thereby blunting the Warburg effect and slowing tumor progression(Du et al. 2011; Yu et al. 2013; Abril et al. 2021; Kumar And Lombard 2018; Zhu et al. 2021; Hu et al. 2021). Similarly, researchers have shown that inhibiting malonyl-CoA catabolism can damage cancer cells: blocking malonyl-CoA decarboxylase (MCD) causes malonyl-CoA to accumulate, which in turn increases protein malonyl-CoA levels. In breast cancer models, the pharmacological inhibition of MCD created toxic conditions for cancer cells and was identified as metabolically vulnerable (Zou et al. 2023; Sambandam et al. 2004). Essentially, forcing cancer cells to experience hypermalonylation (either by SIRT5 inhibition or malonyl-CoA accumulation) can tip their finely tuned metabolism into dysfunction, leading to growth arrest or cell death. This tumor-suppressive role of Kmal is being actively explored; it represents a novel way to target the metabolic fragility of cancer cells.

On the other hand, certain malonylation events or contexts might promote tumorigenesis, illustrating the duality of Kmal. Cancer is a disease characterized by dysregulated signaling and gene expression as well as metabolism. If malonylation occurs on a tumor suppressor protein or a DNA-repair enzyme and inactivates it, it could favor tumor development. Although research in this area is nascent, one study in neurofibromatosis type 2 (NF2) – a tumor syndrome – indicated malonylation’s involvement in lipid metabolic changes that support tumor growth (Zou et al. 2023). In NF2-deficient cells, malonyl-CoA accumulation and malonylation of CPT1C (an ACC-regulated enzyme in the brain) are linked to aberrant mTOR signaling and lipid accumulation, potentially fostering a tumor-permissive environment (Table 2). More direct evidence of malonylation aiding tumors comes from the flipside of the SIRT5 studies: cancer cells *actively exploit* SIRT5 to remove malonylation, implying that if malonylation is left unchecked, it would harm them. In other words, cancer cells treat malonylation as something to eliminate. This is exemplified in acute myeloid leukemia (AML), where SIRT5 was identified as a critical metabolic effector. Chen et al. (2021) reported that AML cells are “addicted” to SIRT5; they require SIRT5 to continuously remove malonyl and succinyl groups that otherwise accumulate on metabolic enzymes and slow proliferation (Yan et al. 2021). Disabling SIRT5 in AML (through CRISPR or RNAi) leads to a failure of leukemic cells to proliferate and induce apoptosis while having relatively minor effects on normal hematopoietic cells (Yan et al. 2021; Juszczak et al. 2024). This differential sensitivity positions SIRT5 as a promising therapeutic target in cancer. A novel SIRT5 inhibitor named NRD167 was shown to effectively inhibit SIRT5’s demalonylase activity in leukemia cells, causing an increase in protein malonylation and a corresponding block in leukemic growth (Li And Melnick 2021). In mouse models of AML, treatment with SIRT5 inhibitors or genetic ablation of SIRT5 significantly delays leukemia progression and extends survival, confirming that SIRT5 (and, by extension, protein malonylation) is a drug target (Yan et al. 2021). In addition to AML, SIRT5 overexpression has been reported in liver cancer, colorectal cancer, and other cancers and is often correlated with poor prognosis, suggesting that many tumors benefit from suppressing malonylation. In these contexts, malonylation itself acts as a tumor-suppressive force that the cancer must overcome. Thus, therapies that increase malonylations in tumor cells (via SIRT5 inhibition, MCD inhibition, or the delivery of cell-permeable malonyl-CoA analogs that specifically malonylate certain targets) are being explored as anticancer strategies. Conversely, it is conceivable that in some cases, we may want to reduce malonylation, for example, if a particular malonylation event is found to activate an oncogenic pathway. However, the current evidence suggests that increased global malonylation is unfavorable for cancer cells, whereas the cancer-promoting roles of Kmal are likely nuanced and specific to certain proteins or stages.

In summary, lysine malonylation has a bifunctional influence on cancer. Its overall increase (such as when SIRT5 is absent) tends to impair the metabolic flexibility and growth of cancer cells, a suppressive effect that researchers are trying to harness for therapy (Zou et al. 2023). On the other hand, cancer cells have evolved ways to keep malonylation in check, underscoring that uncontrolled malonylation is detrimental to them. The emerging therapeutic view is that SIRT5 inhibition or malonyl-CoA modulation could selectively target the metabolic fragility of cancer cells, a strategy strengthened by consistent observations across metabolic (Du et al. 2011; Tan et al. 2014), inflammatory (Duan et al., 2025), renal (Baek et al. 2023), and cardiac diseases (Wei et al. 2024; She et al. 2023). The development of SIRT5 inhibitors (for example, NRD167) now provides a tool to intentionally increase malonylation in tumors, and early results in both leukemia and solid tumors (such as lung cancer) are promising (Zou et al. 2023; Li And Melnick 2021). Moving forward, a deeper understanding of specific Kmal sites that affect oncogenic or tumor suppressor pathways is important. This could reveal cases where malonylation acts positively (e.g., malonylation activating a proapoptotic factor would be good, whereas malonylation inactivating a tumor suppressor would be bad). With such knowledge, combination therapies might be devised, for example, the use of a SIRT5 inhibitor to broadly increase malonylation coupled with another drug to counter any protumor malonylation effects. Finally, malonylation levels or SIRT5 expression might serve as biomarkers for stratifying cancers that respond to these metabolic interventions. In essence, lysine malonylation adds a new layer to our understanding of cancer metabolic reprogramming, offering both insights into tumor biology and tangible avenues for treatment.

## Broader perspectives and future directions

### Interplay with other PTMs

Lysine malonylation does not act in isolation; it is part of a rich repertoire of PTMs that collectively regulate proteins. An individual lysine residue can be a hotspot for multiple modifications (acetylation, methylation, ubiquitination, succinylation, etc.), and these modifications can influence one another (either by steric competition or by sequential/conditional layering). The existence of more than twenty distinct lysine PTMs in cells, many of which are carbon-based acylations such as Kmal, underscores the importance of this crosstalk (Zou et al. 2023; Wang And Cole 2020). For example, acetylation (which adds a neutral acetyl group) and malonylation (which adds a negatively charged group) of the same protein have very different effects on its charge and function. In mitochondrial metabolism, enzymes such as *pyruvate dehydrogenase* or *succinate dehydrogenase* carry multiple acylations, suggesting that the cell may use different acylation “codes” to fine-tune enzyme activity in response to nutrient status (Du et al. 2011; Colak et al. 2015; Dreute et al. 2025). Malonylation and succinylation, in particular, often occur under similar high-nutrient conditions (since malonyl-CoA and succinyl-CoA accumulate when the Krebs cycle slows), and both add acidic groups to lysines. These modifications can have additive or redundant effects; indeed, the role of SIRT5 as a dual demalonylase/desuccinylase implies coordinated control of these marks (Du et al. 2011; Colak et al. 2015; Lanouette et al. 2014). Crosstalk also occurs between malonylation and phosphorylation or ubiquitination. For example, if malonylation of a lysine prevents a kinase from phosphorylating a nearby serine (due to a conformational change), it can indirectly affect signaling cascades. Conversely, signaling events might change metabolism in ways that alter malonyl-CoA levels and thus Kmal. Another layer of interplay involves histones and transcription factors: while lysine acetylation of histones is well known to promote a relaxed chromatin state and active transcription, lysine malonylation of histones has been less explored. However, given its negative charge, one could hypothesize that histone malonylation, such as acetylation, reduces histone‒DNA affinity and might activate gene expression, perhaps specifically in metabolic or stress‒responsive genes. Recent studies on other *negatively charged acylations* (such as succinylation and crotonylation) have shown that they can significantly impact gene expression programs. It will be interesting to see if malonylation works in concert or antagonistically with these modifications on chromatin (for example, does malonylation of a histone mark the same active enhancers that are crotonylated or does it mark distinct regions?). As tools improve, we expect to map these combinatorial PTM patterns with high resolution. This systems-level view will clarify how malonylation fits into broader regulatory networks, whether as a primary switch or as a fine-tuner that reinforces signals from other PTMs. Understanding this interplay is not merely academic; it can reveal synergistic targets for therapy (e.g., a combination of a deacetylase inhibitor and a demalonylase inhibitor may more effectively perturb cancer cell metabolism than either alone).

### Regulatory mechanisms and therapeutic potential of lysine malonylation

The precise regulation of lysine malonylation (Kmal) is orchestrated by specialized enzymes and cofactors, which dynamically modulate its addition and removal. Among these regulatory entities, sirtuin 5 (SIRT5) and its metabolite malonyl-CoA serve as central regulators, influencing diverse physiological processes ranging from cardiac metabolism to tumor biology (Wu et al. 2024; Li et al. 2024). Understanding these regulators and their interactions with other posttranslational modifications (PTMs) provides novel therapeutic opportunities in metabolic, inflammatory, and oncological diseases (Yan et al. 2021; Fabbrizi et al. 2023; Fiorentino et al. 2022; Folmes And Lopaschuk 2007; Rajabi et al. 2022) (Table 3). Recent studies have expanded this regulatory landscape. For example (Duan et al. 2025), demonstrated that malonate-driven protein malonylation enhances CD8⁺ T-cell memory, directly linking Kmal to adaptive immunity. In skeletal and cartilage biology (Ke et al. 2024) and (Liu et al. 2025a, b), showed that SIRT5-dependent regulation of β-catenin and chondrocyte metabolism influences osteogenesis and osteoarthritis development. In cardiology (Wei et al. 2024), reported that GSTP1 demalonylation by SIRT5 is a mechanism that suppresses pyroptosis in diabetic cardiomyopathy. In renal disease (Baek et al. 2023), revealed that SIRT5 limits nonmitochondrial malonylation in diabetic kidney disease, thereby preserving renal function. Finally (Zhang et al. 2023a, b), revealed SIRT5/KAT2A-mediated control of histone malonylation, adding an epigenetic dimension to Kmal regulation. Collectively, these studies highlight how Kmal is a convergent mechanism across immunity, metabolism, bone biology, the kidney, and epigenetics.

**Table 3:** Regulatory enzymes, cofactors, and disease associations associated with lysine malonylation

Regulatory Enzyme/Cofactor	Function/Role	Associated Diseases	Therapeutic Targets/Interventions	Key Proteins/Pathways Affected	References
SIRT5	NAD++-dependent demalonylase/desuccinylase; regulates mitochondrial and cytosolic malonylation	Diabetic cardiomyopathy, osteoarthritis, cancer	SIRT5 agonists (e.g., MC3138), SIRT5 inhibitors (e.g., suramin analogs)	ANT2, CPT2, p53, chondrocyte metabolic enzymes	(Wu et al. 2024; Li et al. 2024)
Malonyl-CoA	Substrate for malonylation; synthesized by ACC1, degraded by MCD. Links glucose/lipid metabolism	Metabolic syndrome, sepsis, renal cell carcinoma	ACC inhibitors (e.g., ND-630), MCD activators	GAPDH, VDAC2, PKM2, SDH	(Zhou et al. 2023; Bowman And Wolfgang 2019)
Malonyl-CoA Decarboxylase (MCD)	Degrades malonyl-CoA to acetyl-CoA; regulates fatty acid oxidation and energy balance	MLYCDD (cardiomyopathy), renal cell carcinoma	MCD gene therapy, small-molecule MCD inhibitors	CPT1, lipid metabolism enzymes, mTORC1 pathway	(Zhou et al. 2023; Monda et al. 2023)
Acetyl-CoA Carboxylase (ACC1)	Synthesizes malonyl-CoA; inhibits fatty acid oxidation via CPT1	Obesity, type 2 diabetes, NAFLD	ACC inhibitors (e.g., ND-630)	CPT1, FASN (fatty acid synthase)	(Folmes And Lopaschuk 2007)
GAPDH	Malonylated in macrophages; promotes TNFα translation during inflammation	Sepsis, chronic inflammatory disorders	SGLT2 inhibitors (e.g., empagliflozin)	TNFα, NF-κB pathway	(Galván-Peña et al. 2019)
VDAC2	Malonylation disrupts mitochondrial calcium flux, inducing ferroptosis in sepsis	Sepsis-induced myocardial dysfunction (SIMD)	Malonyl-CoA synthesis inhibitors	GPX4, mitochondrial permeability transition pore	(She et al. 2023)
CPT2	Desuccinylated by SIRT5; enhances fatty acid oxidation in cardiomyocytes	Diabetic cardiomyopathy	SIRT5 activators	Mitochondrial fatty acid transport, lipid metabolism	(Wu et al. 2024)

*ACC1*, Acetyl-CoA carboxylase 1; *ANT2*, Adenine nucleotide translocase 2; *CPT1/2*, Carnitine palmitoyltransferase 1/2; *GAPDH*, Glyceraldehyde-3-phosphate dehydrogenase; *VDAC2*, Voltage-dependent anion channel 2; *MLYCDD*, Malonyl-CoA decarboxylase defic iency; *NAFLD*, Nonalcoholic fatty liver disease

Key Pathways: Fatty Acid Oxidation (FAO): Regulated by MCD and CPT2. mTORC1: Influenced by malonyl-CoA levels via MCD. NF-κB: Activated by malonylated GAPDH in macrophages

Therapeutic Strategies: SIRT5 Agonists: Improve mitochondrial function in metabolic diseases. MCD Activators: Counteract lipid accumulation in cancer. ACC Inhibitors: Reduce malonyl-CoA synthesis to alleviate insulin resistance

### Therapeutic strategies targeting Kmal

Targeting lysine malonylations for therapeutic benefit can be approached from multiple angles: manipulating the “writers,” “erasers,” or the supply of the malonyl donor. One straightforward strategy is to modulate SIRT5, the chief Kmal eraser. In diseases where excessive malonylation is harmful (such as sepsis or diabetes), activating SIRT5 or increasing its expression could remove malonyl groups from critical proteins. Small-molecule SIRT5 activators are not yet widely available, but research into sirtuin-activating compounds (STACs) for SIRT1 provides a template for discovering compounds that might increase SIRT5 activity or affinity for certain substrates. Another approach is gene therapy or small interfering RNA (siRNA) to increase SIRT5 in specific tissues; for example, liver-targeted gene therapy delivering *SIRT5* could treat fatty liver disease or type 2 diabetes by globally reducing malonylation of hepatic metabolic enzymes (mirroring the effect of SIRT5 overexpression that improved metabolic health in obese mice) (Zou et al. 2023). Conversely, in cancer or diseases where we want to *increase* malonylation, SIRT5 inhibitors play a role. As discussed, SIRT5 inhibitors such as NRD167 show promise in preclinical cancer studies. These inhibitors essentially “lock” malonylation (and succinylation) on proteins by preventing their removal. A potential side effect is the concomitant accumulation of succinylation and glutarylation (since SIRT5 also removes those (Colak et al. 2015), but this broad effect might actually be advantageous in targeting cancer metabolism. The selectivity of SIRT5 inhibitors is an active area of research; the goal is to avoid off-target effects on other sirtuins (SIRT1-4,6,7) to minimize unrelated toxicity. Encouragingly, SIRT5-knockout mice are largely healthy, indicating that transient inhibition of SIRT5 in adults might be tolerated, providing a reasonable therapeutic window.

Aside from sirtuins, the “writers” of malonylation are less defined, but one likely class is lysine acetyltransferases (KATs), which can use acyl-CoA substrates promiscuously. Enzymes such as p300/CBP have been shown to catalyze lysine propionylation and butyrylation via the corresponding acyl-CoAs; it is conceivable that at high malonyl-CoA levels, p300 or related KATs might also catalyze lysine malonylation. If specific malonyltransferases are identified, they also become drug targets; inhibitors of those enzymes could reduce Kmal on particular substrates. For example, if a certain KAT is responsible for malonylating a mitochondrial protein involved in apoptosis, inhibiting KAT might promote the apoptosis of cancer cells. At present, however, no dedicated lysine “malonylase” (writer) has been conclusively isolated; many researchers suspect that a significant portion of malonylations may occur nonenzymatically when the malonyl-CoA thioester reacts with lysine (especially in the high-pH environment of mitochondria). This nonenzymatic aspect means that controlling substrate availability (malonyl-CoA) is crucial. Therefore, another therapeutic strategy is to manipulate malonyl-CoA metabolism. We have two key enzyme targets in this regard: ACC (acetyl-CoA carboxylase), which creates malonyl-CoA, and MCD (malonyl-CoA decarboxylase), which destroys malonyl-CoA. Under conditions of malonyl-CoA excess (e.g., obesity, cardiac ischemia, sepsis), inhibiting ACC or activating MCD can decrease malonyl-CoA, thereby lowering malonylation. ACC inhibitors (such as ND-630, also known as the *drug candidate PF-05175157*) have been tested for the treatment of metabolic diseases; these inhibitors are predicted to reduce Kmal broadly. Interestingly, ND-630 was used in a sepsis heart study and significantly protected the heart by lowering malonyl-CoA and VDAC2 malonylation (She et al. 2023). This finding highlights how an existing metabolic drug can be repurposed to influence a PTM. Conversely, in cancer therapy, one might inhibit MCD (thereby increasing malonyl-CoA and Kmal) to harm tumor cells, as demonstrated by the toxic effect of MCD inhibition on breast cancer cell viability (Zou et al. 2023). Indeed, MCD inhibitors can synergize with SIRT5 inhibitors, both pushing cells toward hypermalonylation from different angles (one increasing the mark’s addition, the other preventing its removal). However, care must be taken, as these interventions can also affect normal cells; thus, tumor-targeted delivery systems (such as nanoparticle carriers or tumor-specific promoters) might be needed to concentrate the effects in cancer cells. In addition to enzymes, small-molecule modulators that directly affect the malonylation of a specific protein could be envisioned. For example, if GAPDH malonylation at K213 is a major driver of inflammation, a small molecule that shields that lysine or competes with malonyl-CoA for binding could be anti-inflammatory. There is preliminary work on peptide-based inhibitors that mimic the lysine substrate and trap acyltransferases or sirtuins, which could be customized for malonylation pathways in the future. Additionally, dietary interventions (like a high-fat ketogenic diet that raises ketones and lowers malonyl-CoA, akin to SGLT2 inhibitor effects) or supplements (like biotin, which influences ACC activity) might offer supportive ways to modulate malonylation in less acute scenarios.

### Challenges and future research directions

While targeting lysine malonylation is conceptually attractive, there are significant challenges and open questions. One challenge is specificity; many of the tools that alter malonylation (e.g., SIRT5 inhibitors and ACC/MCD modulators) also affect other pathways and PTMs. For example, increasing SIRT5 activity might eliminate not only malonylation but also succinylation, potentially blunting beneficial signaling pathways that rely on succinylation. Conversely, SIRT5 inhibition in a patient could lead to the accumulation of various acyl modifications beyond malonylation, with unpredictable outcomes. Therefore, a deeper understanding of substrate selectivity is needed: are there malonylation sites that can be targeted without globally affecting cell metabolism? Drug delivery and timing constitute another concern; for example, in sepsis, one would need to intervene quickly to prevent organ damage via malonylation, so any therapeutic has to act quickly in the metabolic state. In cancer, long-term suppression of SIRT5 might induce compensatory pathways (cells could upregulate other deacetylases or adapt their metabolism). Thus, combination therapies and careful monitoring are key. On the basic research front, identifying bona fide lysine malonyl-transferases remains a priority, as it will fill a major gap in the enzymatic map of this PTM. Additionally, the development of high-quality malonyl-lysine antibodies for specific sites could enable simpler detection (e.g., an ELISA for malonylated talin-1 as an ESRD biomarker or immunohistochemistry for malonylated histone H3 in cancers). Another challenge is the paradoxical, tissue-dependent role of Kmal. For example, Duan et al. (2025) reported that protein malonylation promotes CD8⁺ T-cell memory formation, highlighting a beneficial role in immunity, whereas (Ke et al. 2024) and (Liu et al. 2025a, b) demonstrated that excessive malonylation in osteoblasts and chondrocytes contributes to impaired bone formation and osteoarthritis progression. Similarly (Wei et al. 2024), reported that SIRT5-mediated demalonylation of GSTP1 protects against pyroptosis in diabetic cardiomyopathy, whereas (Baek et al. 2023) reported that SIRT5 restrains nonmitochondrial malonylation in diabetic kidney disease (Zhang et al. 2023a, b). further added an epigenetic perspective, showing that histone malonylation is dynamically regulated by SIRT5 and KAT2A. Together, these studies illustrate that malonylation can either sustain cellular health or drive pathology depending on the tissue and context. For future directions, one exciting area is the exploration of malonylation in contexts such as neurodegenerative diseases and aging. The brain has unique lipid metabolism pathways (malonyl-CoA is key in appetite control via CPT1C in the hypothalamus) (Table 2), and the role of malonyl-CoA in neuronal function or neuroinflammation is yet unknown. Another area is infectious diseases: some pathogens may exploit host malonylation; for example, *Mycobacterium tuberculosis* alters host metabolism in macrophages, and it would be intriguing to see if malonylation of host or bacterial proteins is involved. Given the connection to inflammation, malonylation might also intersect with autoimmune conditions or aging-related inflammation (“inflammaging”). As technology progresses, we anticipate more sensitive mass spectrometry methods that can quantify malonylation occupancy on proteins in clinical samples and CRISPR-based tools to edit specific lysines to nonmalonylatable analogs (e.g., lysine to arginine mutations in cell models) to definitively test the function of each malonylation site. In conclusion, lysine malonylation has moved to the forefront as a significant PTM in cellular regulation. It serves as a nexus between metabolism and cell signaling, with demonstrated relevance in metabolic disorders, inflammatory pathways, reproductive biology, kidney disease, and cancer. Future research should aim to resolve the context-specific roles of Kmal across tissues and diseases, distinguishing when it acts as a protective regulator versus a pathogenic driver. By continuing to dissect its mechanisms and develop ways to manipulate it, we stand to identify innovative diagnostic markers and therapies for a range of conditions. As one recent review highlighted, malonylation represents a promising diagnostic and therapeutic target, and advancing methods to identify and modulate malonylation will be crucial for translating these discoveries into clinical applications (Zou et al. 2023). The coming years will likely witness malonylation transitioning from a biochemical curiosity to a mainstream target in precision medicine, reflecting our growing mastery of the “epigenetics of metabolism.”

## Conclusion

Lysine malonyl (Kmal) has emerged as a key metabolic regulator that influences diverse biological processes, such as energy metabolism, inflammation, and tumor progression. Its regulation by malonyl-CoA and SIRT5 underscores its dynamic role in cellular homeostasis and disease pathogenesis. In metabolic disorders such as diabetes and obesity, Kmal contributes to insulin resistance and lipid accumulation, whereas in inflammatory diseases, it modulates immune responses through cytokine translation. Its dual role in cancer highlights both tumor-suppressive and pro-oncogenic functions, positioning Kmal as a potential therapeutic target. Advances in proteomics have provided insight into its regulatory mechanisms, but further research is needed to delineate tissue-specific malonylation patterns and their interactions with other PTMs. Targeting Kmal through SIRT5 modulators, malonyl-CoA metabolism regulators, and PTM-specific inhibitors represents a promising avenue for disease intervention. As our understanding increases, Kmal could serve as both a biomarker and a therapeutic target in metabolic, inflammatory, and oncological diseases. Future studies should prioritize precision medicine approaches that leverage Kmal’s regulatory network to develop targeted therapies for improved clinical outcomes.

## Data Availability

No datasets were generated or analysed during the current study.
